# Allele-Specific MicroRNA-Mediated Regulation of a Glycolysis Gatekeeper PDK1 in Cancer Metabolism

**DOI:** 10.3390/cancers13143582

**Published:** 2021-07-17

**Authors:** Sugarniya Subramaniam, Varinder Jeet, Jennifer H. Gunter, Judith A. Clements, Jyotsna Batra

**Affiliations:** 1 School of Biomedical Sciences, Faculty of Health, Institute of Health and Biomedical Innovation, Queensland University of Technology, Brisbane 4000, Australia; ssugarniya@gmail.com (S.S.); varinder.jeet@mq.edu.au (V.J.); jennifer.gunter@qut.edu.au (J.H.G.); j.clements@qut.edu.au (J.A.C.); 2 Australian Prostate Cancer Research Centre-Queensland (APCRC-Q), Translational Research Institute, Queensland University of Technology, Woolloongabba 4102, Australia

**Keywords:** PDK1, metabolic reprogramming, single-nucleotide polymorphisms, microRNAs

## Abstract

**Simple Summary:**

MicroRNAs are small non-coding RNAs that regulate gene expression. Single Nucleotide Polymorphisms (SNPs), which have been previously associated with cancer risk may interfere with the function of microRNAs by changing the binding of microRNA to its target gene. We sought to identify the role of prostate cancer risk associated SNPs in regulating a vital enzyme of cancer metabolism, pyruvate dehydrogenase kinase 1 (PDK1) via microRNAs. The microRNA and gene interaction was studied using computational predictions and by various laboratory assays. Our study highlighted the role of PDK1 and its regulation by microRNAs in cancer metabolism and disease progression. The findings from this study suggest that analysis of microRNAs and their interactions with SNPs could provide valuable insights into the complicated mechanisms of prostate cancer risk associated SNPs and identify suitable moleculer pathways for targeted therapy.

**Abstract:**

Background: Emerging evidence has revealed that genetic variations in microRNA (miRNA) binding sites called miRSNPs can alter miRNA binding in an allele-specific manner and impart prostate cancer (PCa) risk. Two miRSNPs, rs1530865 (G > C) and rs2357637 (C > A), in the 3′ untranslated region of pyruvate dehydrogenase kinase 1 (PDK1) have been previously reported to be associated with PCa risk. However, these results have not been functionally validated. Methods: In silico analysis was used to predict miRNA–PDK1 interactions and was tested using PDK1 knockdown, miRNA overexpression and reporter gene assay. Results: PDK1 expression was found to be upregulated in PCa metastasis. Further, our results show that PDK1 suppression reduced the migration, invasion, and glycolysis of PCa cells. Computational predictions showed that miR-3916, miR-3125 and miR-3928 had a higher binding affinity for the C allele than the G allele for the rs1530865 miRSNP which was validated by reporter gene assays. Similarly, miR-2116 and miR-889 had a higher affinity for the A than C allele of the rs2357637 miRSNP. Overexpression of miR-3916 and miR-3125 decreased PDK1 protein levels in cells expressing the rs1530865 SNP C allele, and miR-2116 reduced in cells with the rs2357637 SNP A allele. Conclusions: The present study is the first to report the regulation of the PDK1 gene by miRNAs in an allele-dependent manner and highlights the role of PDK1 in metabolic adaption associated with PCa progression.

## 1. Introduction

Prostate cancer (PCa) is one of the most common malignancies and a major cause of cancer-related deaths in men [1]. Prostate tumour cells undergo metabolic reprogramming to accelerate glycolysis, even in the presence of abundant oxygen, while shifting energy production away from mitochondrial oxidative phosphorylation (OXPHOS), a process referred to as aerobic glycolysis. Increased rates of glycolysis are widely recognised as one of the hallmarks of aggressive cancers, including PCa [2,3]. The prostate has a specific metabolic profile that changes during prostatic disease from prostate intraepithelial neoplasia (PIN) to localised PCa to metastasis. In differentiated prostate epithelial cells, citrate is the final product of glucose metabolism. During the initial steps of tumourigenesis, OXPHOS is active, and cancer cells adapt to the Warburg effect only in the metastatic or castration-resistant stages in PCa [4]. The elevated level of glycolysis is required to meet the enhanced energy requirements of rapidly dividing tumour cells and also confers cancer cells with metastatic advantage by promoting other oncogenes such as pyruvate dehydrogenase kinases (PDKs), hexokinase 2 and hypoxia-inducible factor-1α (HIF-1α) [5,6]. The fate of pyruvate into the tricarboxylic acid cycle (TCA) cycle or glycolysis is regulated by pyruvate dehydrogenase (PDH), which catalyses the conversion of pyruvate to acetyl-coenzyme A and regulates its entry into the TCA cycle. Inactivation of PDH leads to a reduction in the oxidation of pyruvate in the mitochondria and increases the conversion of pyruvate to lactate in the cytoplasm [7]. PDH activity is negatively regulated through phosphorylation by PDKs, including PDK1. PDKs are overexpressed in various cancers, including PCa, and contribute to high glycolytic activity in cancer cells [8,9]. High glycolytic activity permitted by PDKs leads to increased tumour growth, migration, invasion and immune resistance in various cancers [10,11,12].

In many tumour cells, the expression of glycolytic enzymes is induced by hypoxia by activating HIF-1α. PDKs are a direct target of HIF-1α, which modulates metabolism through early glycolytic shift [13]. Under hypoxic conditions, tumour cells acquire many malignant properties correlated with increased risk of metastasis and mortality [14,15].

miRNAs are small non-coding RNAs of approximately 22 nucleotides. They regulate gene expression by binding to their complementary sites within the 3′-UTRs of target mRNAs resulting in mRNA translational repression or transcript degradation [16,17]. miRNAs regulate the glycolytic activity of cancer cells by directly targeting key enzymes or transporters of the glycolytic pathway and multiple oncogenic signalling pathways [18,19]. Studies have indicated that PDK1 is indirectly regulated by miRNAs in various cancers [20]. Genetic variations within the miRNA target genes, referred to as miRSNPs, may affect the regulatory effect of miRNAs to their target genes, thus contributing to tumourigenesis [21,22]. Previously, a large-scale miRSNP association study by Stegeman et al. reported two PDK1 miRSNPs in the 3′ untranslated region (3′UTR), namely, rs1530865 (G > C) with an OR= 0.8 (0.75–0.86), *p* = 2.28 × 10^−9^, and rs2357637 (C > A) with an OR= 0.81 (0.76–0.88), *p* = 3.66 × 10^−8^, which are associated with the risk of PCa [23]. However, no studies have been conducted on miRNA–SNP interaction in the PDK1 gene. In this study, we validated the role of PDK1 in PCa metabolism and the association between miRNAs and miRSNPs in PCa.

## 2. Results

### 2.1. PDK1 Expression is Upregulated with Prostate Tumourigenesis

**Figure 1:**
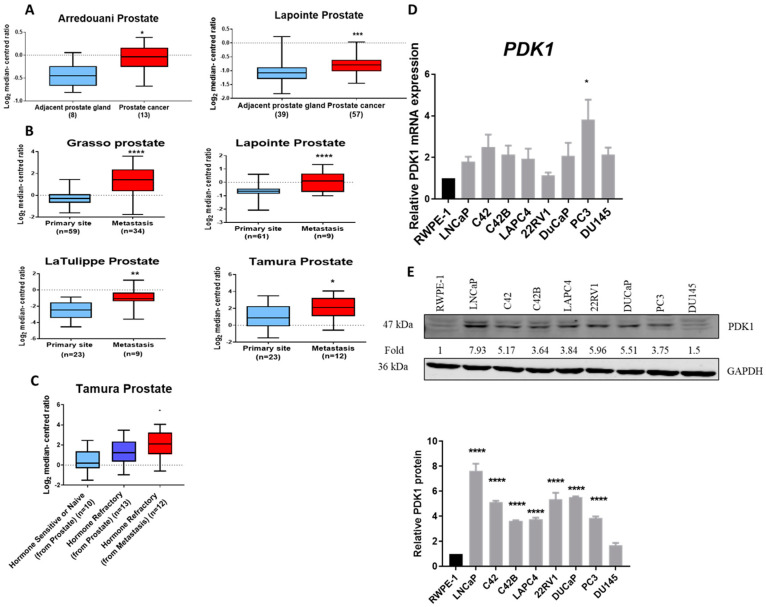
Increased PDK1 expression with disease progression. (**A**) Analysis from the Oncomine database indicated that PDK1 mRNA expression was upregulated in prostate carcinoma compared to the primary site in the Arredounai dataset ((adjacent prostate gland (*n* = 8), prostate cancer (*n* = 13)) and Lapointe dataset ((adjacent prostate gland (*n* = 39), prostate cancer (*n* = 57)). (**B**) PDK1 mRNA expression levels were significantly upregulated in metastasis compared with the primary site in four independent datasets (Grasso (primary site (*n* = 59), metastasis (*n* = 34)), Lapointe (primary site (*n* = 61), metastasis (*n* = 9)), La Tulippe (primary site (*n* = 23), metastasis (*n* = 9)) and Tamura (primary site (*n* = 23), metastasis (*n* = 12)). (**C**) Higher PDK1 mRNA was observed in patients with hormone refractory compared to naive. Tamura (hormone sensitive (*n* = 10), hormone refractory from prostate (*n* = 13), hormone refractory from metastasis (*n* = 12)). (**D**) PC3 cells showed the highest PDK1 mRNA expression, which was significantly higher when compared to a cell line derived from normal prostate epithelial cells, RWPE-1. (**E**) PDK1 protein was upregulated in prostate cancer cell lines compared to RWPE1 cells. Statistically significant differences were assessed using an unpaired *t*-test (**A**,**B**), and one-way ANOVA with Tukey’s multiple comparison test (**C**–**E**) (**** *p* < 0.0001, *** *p* < 0.001, ** *p* < 0.01, * *p* < 0.05).

To assess PDK1 expression in clinical PCa specimens, gene expression analysis was performed using the Oncomine webserver. PDK1 mRNA expression was upregulated in PCa compared to the adjacent prostate gland in two independent datasets: Arredounai (adjacent prostate gland (*n* = 8), prostate cancer (*n* = 13)) and Lapointe (adjacent prostate gland (*n* = 39), prostate cancer (*n* = 57)) (Figure 1A). Further, PDK1 was significantly upregulated in metastasis compared to the primary site in four independent datasets—Grasso (primary site (*n* = 59), metastasis (*n* = 34)), Lapointe (primary site (*n* = 61), metastasis (*n* = 9)), La Tulippe (primary site (*n* = 23), metastasis (*n* = 9)) and Tamura (primay site (*n* = 23), metastasis (*n* = 12)) (Figure 1B)—and in hormone-refractory metastatic tissue compared with hormone-sensitive and hormone-refractory prostate tissue (Figure 1C). Further, gene analysis was performed from the Cancer Genome Atlas (TCGA). Analyses of PDK1 expression in normal solid tissues (*n* = 52), primary tumour (*n* = 497) and metastasis (r) in the TCGA microarray dataset did not show any significance in PDK1 expression (Supplementary Figure S1). Next, we evaluated the expression of PDK1 in multiple prostate cell lines. The metastatic cell line, PC3, showed the highest expression of PDK1 mRNA compared to the cell line derived from normal prostate epithelial cells, RWPE-1 (Figure 1D). Although PDK1 mRNA was elevated in prostate cancer cell lines- LNCaP, C42, C42B, LAPC4, 22RV1, DUCaP and DU145 compared with the RWPE1 cellsthe effect was not significant. However, the protein level of PDK1 was significantly higher in all PCa cell lines (LNCaP, C42, C42B, LAPC4, 22RV1, DUCaP and PC3) compared to RWPE-1 (Figure 1E), except DU145 cells.

### 2.2. PDK1 Knockdown Does Not Alter Proliferation but Increases Prostate Cancer Cell Migration and Invasion

**Figure 2:**
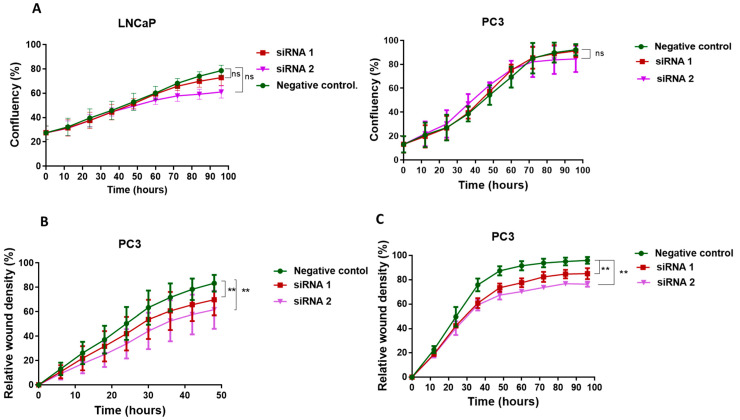
PDK1-mediated proliferation, migration and invasion of prostate cancer cells. (**A**) PDK1 knockdown did not exert a significant effect on prostate cancer cell (LNCaP and PC3) proliferation as measured by the IncuCyte cell imaging system. (**B**) Suppression of PDK1 reduced migration of PC3 cells. PC3 cells were grown to confluence, and a wound was created using a 96-well wound maker, and images were recorded over 2 days. The percentage of cells that migrated through the wound was plotted as relative wound density in non-targeting siRNA control vs. PDK1 knockdown cells. (**C**) PC3 cells with PDK1 knockdown showed a reduction in the invasion. Cells were layered onto Matrigel-coated plates, and a wound was created as above. The percentage of cells that invaded through the wound was plotted as relative wound density in negative control vs. PDK1 knockdown cells. Statistically significant differences were assessed using a one-way ANOVA with Tukey’s multiple comparison test; Mean ± SEM, *n* = 3, ** *p* < 0.01.

For subsequent experiments, we selected LNCaP and PC3 cells because these two cell lines were found to express high levels of PDK1. To assess the effects of PDK1 inhibition on these tumour cell lines, two siRNAs were transiently transfected (siRNA 1 and 2) in LNCaP and PC3 cell lines. Significant knockdown efficiency was observed in LNCaP (80%) and PC3 (90%) cells at the mRNA level in siRNA-treated groups as compared to the non-targeting siRNA control (Supplementary Figure S2). Knockdown of PDK1 had no significant effect on the proliferation of LNCaP and PC3 cell lines compared to the non-targeting siRNA control (Figure 2A). This result suggests that PDK1 may not play a role in PCa cell proliferation under the given conditions. Metastasis is a hallmark of cancer, and an initial step in metastasis is migration. Since PDK1 was found to be overexpressed in PCa metastasis, we hypothesised that PDK1 knockdown might reduce the migration of PCa cells. The migratory ability of PC3 cells with the suppression of PDK1 was significantly reduced compared to the non-targeting siRNA control (Figure 2B) (Supplementary Figure S3A). Further, the suppression of PDK1 expression in PC3 cells led to a statistically significant reduction in the number of invading cells through Matrigel (Figure 2C) (Supplementary Figure S3B).

### 2.3. The Effect of PDK1 Suppression on Mitochondrial Metabolism

**Figure 3:**
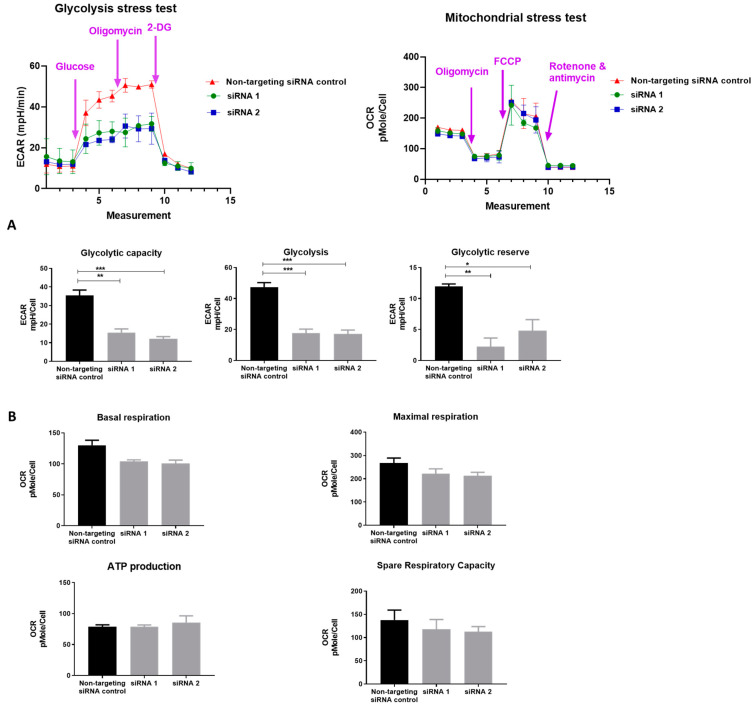
PDK1 knockdown inhibits glycolysis and does not affect mitochondrial respiration in LNCaP cells. (**A**) LNCaP cells were seeded in Seahorse XFe96 cell culture microplates. The cells were treated with 10 nM of siRNAs for 72 h, followed by sequential treatment with glucose (10 mM), oligomycin (ATP synthase inhibitor) (1 μM) and 2-DG (glycolytic inhibitor) (100 mM). Extracellular acidification rate was measured. PDK1 siRNAs reduced all three glycolytic parameters in LNCaP cells. (**B**) Oxygen Consumption Rate (OCR) rate was measured with PDK1 suppression. LNCaP cells were transfected with 10 nM non-targeting siRNA control or PDK1 siRNA. Seventy-two hours after transfection, cells were treated sequentially with oligomycin (ATP synthase inhibitor) (1.2 μM), FCCP (mitochondrial uncoupler) (1 μM), and rotenone plus antimycin A (respiratory chain inhibitors) (1 μM). OCR was measured for each treatment. Mitochondrial respiration was not altered by PDK1 suppression; only proton leak was affected. Statistically significant differences were assessed using a one-way ANOVA with Tukey’s multiple comparison test; data are expressed as Mean ± SEM, ** *p* < 0.01, * *p* < 0.05.

To validate PDK1 knockdown, we examined the effect on glycolysis of PCa cells by measuring the extracellular acidification rate (ECAR) post-knockdown. Compared with the non-targeting siRNA control, siPDK in LNCaP cells showed a significant reduction in glycolytic flux, with a 2–4-fold decrease in glycolysis, glycolytic capacity and glycolytic reserve in LNCaP cells (Figure 3A). Thus, the knockdown of PDK1 led to an overall reduction in ECAR levels in LNCaP cells. These results confirm that PDK1 actively induces glycolytic reprogramming in LNCaP cells through the redirection of pyruvate flux into lactate. siRNA treatment did not affect the basal mitochondrial respiration, maximal respiration, ATP production and spare respiratory capacity of LNCaP cells (Figure 3B). This mitochondrial stress test suggests that overall PDK1 knockdown does not significantly impact key parameters of mitochondrial functions. Altogether, glycolysis and mitochondrial stress test results imply that PDK1 is mainly involved in the reprogramming of glycolysis in PCa cells.

### 2.4. Regulation of PDK1 by Hypoxia in PCa Cells

**Figure 4:**
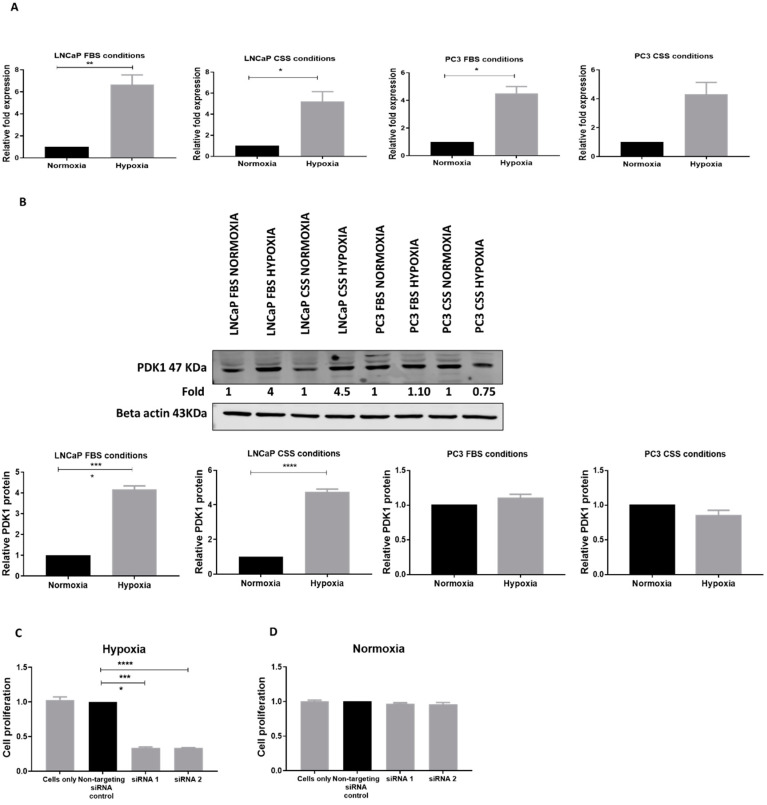
Hypoxia regulated PDK1 in prostate cancer cells. (**A**) PDK1 mRNA expression is significantly upregulated under hypoxic conditions in LNCaP in FBS and CSS conditions and in PC3 cells in FBS conditions. (**B**) PDK1 protein expression is upregulated under hypoxic conditions in LNCaP and no change in PC3 cells in both FBS and CSS conditions. (**C**) Suppression of PDK1 reduced the proliferation of LNCaP under hypoxic conditions. (**D**) Suppression of PDK1 did not affect the proliferation of LNCaP cells under normoxic conditions. The proliferation, as measured by the CyQuant assay, of each group was compared with the non-targeting siRNA control group. Statistically significant differences were assessed using an unpaired *t*-test (**A**,**B**), and one-way ANOVA with Tukey’s multiple comparison test (**C**,**D**); data presented are mean ± SEM, *n* = 3, **** *p* < 0.0001, ** *p* < 0.01, * *p* < 0.05.

Upregulation of glycolytic genes by hypoxia is considered a metabolic adaptation to low oxygen concentration through increased conversion of glucose to lactate [24]. Therefore, we measured PDK1 expression under hypoxic conditions in PCa cells (Figure 4A). LNCaP in both FBS and CSS showed high expression of PDK1 while PC3 showed high expression in FBS under hypoxic compared to normoxic conditions. Comparatively, the androgen-dependent cell line (LNCaP) had the higher expression of PDK1, followed by the androgen-independent cell line (PC3). Further, we investigated PDK1 protein expression in response to hypoxia in LNCaP and PC3 cells. Western blot analysis indicated hypoxia upregulated PDK1 by 4–5 fold in LNCaP cells at the protein level in both FBS and CSS conditions (Figure 4B). However, PC3 cells did not show any changes in PDK1 protein expression in response to hypoxia. This could be due to the higher glycolytic phenotype of PC3 compared to LNCaP cells [25]. These results indicate that PDK1 expression is induced by hypoxia in PCa cells and suggest that cancer cells may produce lactic acid through up-regulation of PDK1 as a metabolic adaptation to hypoxic conditions. To determine the role of PDK1 in metabolic adaptation of LNCaP cells in response to hypoxia, we used siRNAs (siRNA 1 and 2) to suppress PDK1 expression. The CyQUANT assay indicated that the suppression of PDK1 expression significantly reduced the proliferation of LNCaP cells compared to LNCaP cells treated with a non-targeting siRNA control under hypoxic conditions (Figure 4C) but not in normoxic conditions (Figure 4D). This result correlates with the hypothesis that PDK1 is necessary for the proliferation of LNCaP cells under hypoxic conditions. Further, this result indicates that PDK1 is vital for survival for the hypoxic adaptation of PCa cells.

### 2.5. miR-3916-3p and miR-3125 Directly Target the PDK1 rs1530865 miRSNP C Allele and miR-2116 Targets the PDK1 rs2357637 miRSNP A Allele

**Figure 5:**
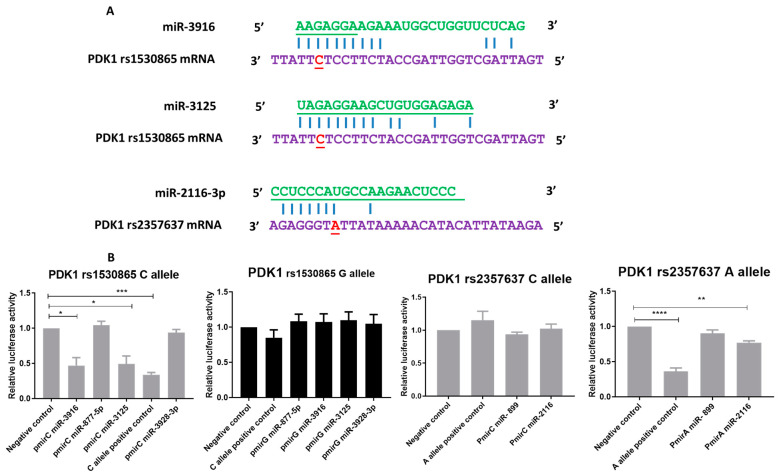
miR-3916-3p and miR-3125 target the PDK1 rs1530865 miRSNP C allele, and miR-2116 targets the PDK1 rs2357637 miRSNP A allele. (**A**) miRNAs miR-3916 and miR-3125 were predicted to interact with the PDK1 rs1530865 miRSNP C allele. Similarly, and miR-2116 predicted to target the rs2357637 miRSNP A allele. Base pairing is shown by a solid vertical line. (**B**) Luciferase reporter assays to measure C/G and C/A allele differential interaction at the PDK1 rs1530865 SNP and PDK1 SNP rs2357637, respectively. LNCaP cells were transfected with C/G and C/A containing reporter constructs and the miRNA mimics. Renilla luciferase activity was normalised to firefly luciferase, and results are presented as a percentage relative to luciferase activity. Data are shown from 3 independent experiments. Reduced luciferase activity observed with the co-transfection of miR-3916, miR-3928 for the rs1530865 miRSNP C allele and miR-2116 with rs2357637 miRSNP A allele. Statistically significant differences were assessed using a one-way ANOVA with Tukey’s multiple comparison test; Mean ± SEM, *n* = 3, *p* < 0.0001 ****, *p* < 0.001 ***, *p* < 0.01 **, *p* < 0.05 *.

Using Mirsnpscore and MicroSNiper computational prediction algorithms, we identified that PDK1 rs1530865 and rs2357637 were predicted to affect the binding of four and two miRNAs, respectively. miR-3916, miR-877, miR-3928 and miR-3125 were predicted to target the PDK1 rs1530865 C allele, and miR-2116 and miR-889 were predicted to target the PDK1 rs2357637 miRSNP A allele (Figure 5A) (Supplementary Figure S4). To validate the miRNA–miRSNP interaction predictions, miRNA–SNP affinity was determined using reporter vector assays. The reporter gene assays showed that the co-transfection of miR-3916 and miR-3125 reduced luciferase activity by 48% and 47%, respectively, for the reporter vector containing the PDK1 UTR with rs1530865 C allele, while not affecting the PDK1 rs1530865 G allele. Further, 69% luciferase reduction was observed with the PDK1 rs1530865 C allele positive control. Similarly, 23% and 66% luciferase reductions, respectively, were observed with miR-2116 and the A allele positive control for the PDK1 rs2357637 A allele compared with the rs2357637 C allele, which is consistent with the in-silico prediction of this miRNA–SNP interaction. There were no luciferase changes observed with miR-877, miR-3928 and miR-889 in any reporter gene assays. This result suggests that miR-3916 and 3125 have a high affinity for the PDK1 rs1530865 C allele and that miR-2116 has an affinity for the PDK1 rs2357637 miRSNP A allele (Figure 5B).

### 2.6. miR-3916, miR-3125 and miR-2116 Reduce PDK1 Protein Levels in Cell Lines with rs1530865 CC SNP Genotype and rs2357637 SNP AA Genotype

We demonstrated that miR-3916 and miR-3125 have a specific affinity for the PDK1 rs1530865 C allele, while miR-2116 has an affinity for PDK1 rs2357637 A allele. To test this allele-specific effect on endogenous mRNA and protein expression, genotyping of various PCa cell lines was performed. All PCa cell lines, LNCaP, PC3, WPMY-1, DuCaP, C4-2B, RWPE-1, VCaP and BPH-1, were homozygous GG for the rs1530865 and CC for the rs2357637 SNPs. Therefore, we sought other cancer cell lines that may have the alternate allele. Testing a range of ovarian cancer cell lines, we observed CAOV3 cells have the GG genotype, OV90 cells have the GC genotype, and OVMZ6 cells have the CC genotype for rs1530865 SNP, while CAOV3, OV90, and OVMZ6 were found to have CC, AC and AA genotypes, respectively, for the rs2357637 miRSNP (Table S1). Thus, only the ovarian cell lines had homozygous and heterozygous genotypes for both miRSNPs.

First, the predicted miRNAs mimics were transfected in LNCaP cells, which were homozygous GG for rs1530865 and CC for rs2357637 SNPs, respectively. Overexpression of miR-3916, miR-3125 and miR-2116 did not affect PDK1 mRNA levels in LNCaP cells compared to the negative control (Supplementary Figure S5A). Further, Western blot analysis indicated that PDK1 protein levels in LNCaP cells were not changed with miR-3916, miR-3125 and miR-2116 overexpression (Supplementary Figure S5B). This result correlates with our reporter vector assay findings, where miR-3916-3p and miR-3125 did not affect luciferase activity with PDK1 rs1530865 miRSNP G allele and miR-2116 did not affect PDK1 rs2357637 miRSNP C allele luciferase vector.

**Figure 6:**
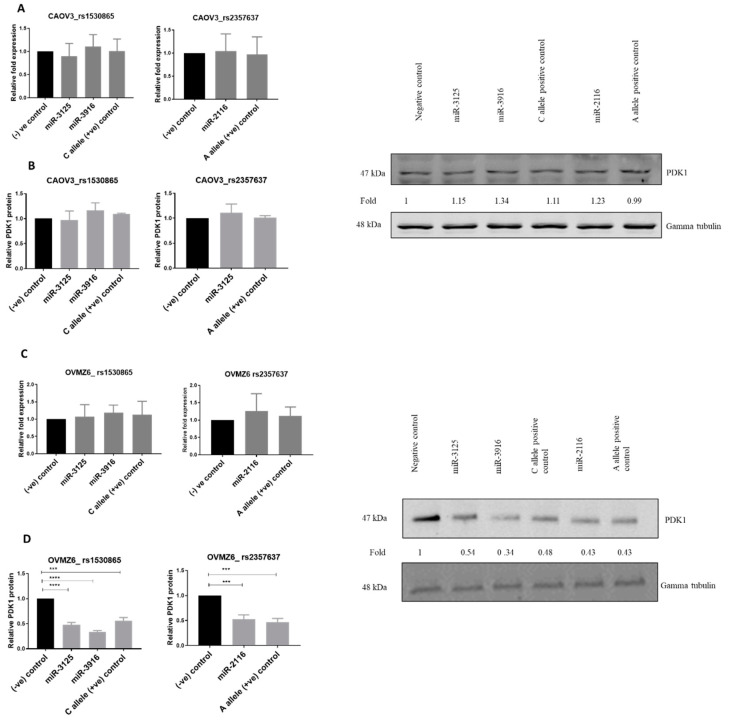
miR-3916, miR-3125 and miR-2116 did not reduce PDK1 mRNA expression and PDK1 protein expression in CAOV3 cells, while miR-3916, miR-3125 and miR-2116 reduced PDK1 protein in OVMZ6 cells. (**A**) Overexpression of miR-3916, miR-3125 and miR-2116 had no effect on PDK1 mRNA level in CAOV3 cells. (**B**) miR-3916, 3125, and miR-2116 did not alter PDK1 protein in CAOV3 cells. (**C**) Overexpression of miR-3916, miR-3125 and miR-2116 had no effect on PDK1 mRNA levels in OVMZ6 cells. (**D**) miR-3916, 3125 and miR-2116 reduced PDK1 protein in OVMZ6 cells. The bar graph in B indicates densitometry analysis of the Western blots. Statistically significant differences were assessed using a one-way ANOVA with Tukey’s multiple comparison test; Mean ± SEM, *n* = 3; a representative Western blot is shown.

Next, miR-3916, miR-3215 and miR-2116 were over-expressed in CAOV3 and OVMZ6 cells to analyse the allele-specific PDK1 regulation by these miRNAs. Overexpression of miR-3916, miR-3125 and miR-2116 did not affect the PDK1 mRNA expression (Figure 6A) and PDK1 protein level (Figure 6B) in CAOV3 cells, which are GG for the rs1530865 genotype and CC for the rs2357637 genotype.

Overexpression of miR-3916, miR-3125, and miR-2116 in OVMZ6 cells did not affect PDK1 mRNA expression (Figure 6C). In contrast, overexpression of miR-3916, miR-3125 and miR-2116 altered the PDK1 protein (Figure 6D) level by 67%, 52% and 47% in OVMZ6 cells, respectively, confirming that miR-3916 and miR-3125 target the C allele of the PDK1 rs1530865 SNP and miR-2116 targets the PDK1 rs2357637 SNP A allele in this cell line. Overall, these data suggest that miR-3916, 3125 and 2116 regulate the PDK1 gene in an allele-dependent manner.

Since we observed allele-dependent regulation of PDK1 in ovarian cancer cells, we examined the expression of PDK1 in patient samples in the Oncomine datasets. Yoshihara (Peritoneum (*n* = 10) ovarian serous adenocarcinoma (*n* = 43)), Tothill (ovarian surface epithelial-stromal tumour (*n* = 18) ovarian carcinoma (*n* = 171)), Anglesio (ovarian surface epithelial-stromal tumour (*n* = 30) ovarian carcinoma (*n* = 44)) and Lu ovarian (ovarian surface epithelium (*n* = 5) ovarian serous adenocarcinoma (*n* = 20)) dataset analysis indicated that PDK1 mRNA expression is up-regulated in ovarian carcinoma (Supplementary Figure S6). This up-regulation of PDK1 in ovarian tumour tissues suggests a potential role of PDK1 in ovarian cancer.

### 2.7. Androgen Regulation of miR-3916 and miR-2116 in LNCaP Cells

**Figure 7:**
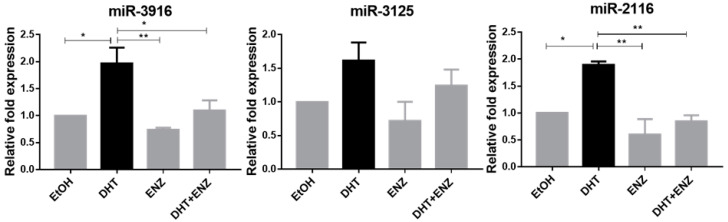
Expression of miR-3916, miR-3125 and miR-2116 in androgen- and anti-androgen-treated LNCaP cells. LNCaP cells were treated with 10 nM of androgen (DHT) and 10 µM of enzalutamide (ENZ) for 48 h. miR-3916 and miR-2116 expression was upregulated with androgen treatment and suppressed with anti-androgen (enzalutamide) treatment. Statistically significant differences were assessed using a one-way ANOVA with Tukey’s multiple comparison test; data presented are mean ± SEM, *n* = 3, ** *p* < 0.01, * *p* < 0.05.

Androgens, by activating the androgen receptor (AR), play an important role in both the development and progression of PCa. Therefore, we evaluated the effect of androgen (DHT) on the expression of miR-3916, miR-3125 and miR-2116 in LNCaP cells. miR-3916 expression and miR-2116 expression were significantly upregulated, but the miR-3125 expression did not change significantly after DHT treatment in LNCaP cells (Figure 7). To determine if this phenomenon was due to AR function, we treated LNCaP cells with enzalutamide, an antagonist of AR. As a result of suppression of a functional AR, the miR-3916 and miR-2116 expression were reduced in the presence of enzalutamide. miR-3125 showed an upward trend with androgen and a downward trend with anti-androgen and a combination of androgen and enzalutamide treatment; however, values were not statistically significant. Overall, these results suggested that androgen signalling pathways regulate miR-3916 and miR-2116 in PCa.

## 3. Discussion

The reprogramming of glycolytic metabolism is vital for tumourigenesis, and various studies have reported that PDK1 plays a major role in the glycolytic metabolism [26,27]. From online datasets, we observed upregulation of PDK1 mRNA expression in both localised and metastatic PCa, suggesting that its expression is increased throughout tumourigenesis. Further, few studies have shown that PDK1 expression is significantly higher in the metastatic tumour than BPH, PIN lesions, and localised prostate tumours [28]. In concordance with this, our results indicate that PDK1 expression is higher in PCa cell lines compared to the normal derived epithelial cell line, RWPE-1. Functional assays with PDK1 suppression indicated that while PDK1 may not play a significant role in the proliferation of PCa cells, the results trended towards lower proliferation rates in PDK1-silenced cells. However, PDK1 has previously been reported to significantly induce the proliferation of non-small cell lung cancer and retinoblastoma [29,30]. Moreover, our results show that PDK1 may play a role in the cell invasion and migration of PCa cells in vitro. Our data are in line with many studies implicating the role of PDK1 in promoting tumour cell migration and invasion in various other cancers [11,29,31,32]. These findings have provided supporting evidence that PDK1 may contribute to the tumour progression and metastasis of PCa.

Notably, in the expression analysis, we observed inconsistent results between the protein and mRNA levels of PDK1. Post-transcriptional and translational regulation could change the protein level of the transcribed mRNA. However, the expression pattern of PDK1 protein in PCa cell lines has shown high expression of PDK1 in both androgen-dependent and androgen-independent metastatic cell lines compared to the normal derived RWPE-1 cell line. The results from this study suggest that PDK1 may be regulated in an androgen-dependent manner in prostate cell lines.

Cancer cells alter their metabolism to promote tumourigenesis, and the common feature of this altered metabolism is an increased Warburg effect [33]. Upregulation of glycolysis leads to tumour microenvironmental acidosis causing the phenotypes which are resistant to acid-induced cell toxicity. Consequently, cell populations with acid resistance promote unconstrained tumourigenesis [34,35]. In PCa, glycolysis is activated in later stages of tumourigenesis [36]. We also observed that PDK1 inhibition significantly reduced glycolysis in a lymph node metastatic cell line, LNCaP. Moreover, PDK1 inhibition did not change ATP production in PCa cells. Lactate is the end product of glycolysis and has been reported to activate HIF-1α, which further activates other genes involved in migration and invasion [37,38]. Our study found that the suppression of PDK1 reduced the glycolysis, migration, and invasion of prostate cancer cells. These results suggest that PDK1 not only plays a role in glycolysis but may contribute to prostate cancer tumourigenesis as well.

Further, we found that PDK1 is upregulated by hypoxia in LNCaP cells. However, we did not see any significant changes with hypoxia in PC3 cells. LNCaP cells are more oxidative phenotype, while PC3 cells are more glycolytic phenotype [25]. Therefore, hypoxic conditions did not change the PDK1 expression in PC3 at the protein level. Moreover, PDK1 suppression did not change the proliferation of LNCaP cells under normoxic conditions while reduced proliferation under hypoxic conditions. This result implies that PDK1 is necessary for the proliferation of LNCaP cells under hypoxic conditions and that PDK1 is essential for cellular adaptation to hypoxia [39], suggesting a possible target for PCa therapeutic intervention.

In this study, two genetic variants in the 3′ UTR of PDK1 were determined to predict whether they contribute to PCa tumourigenesis. The PDK1 3′-UTR contains functional binding sites miR-3916, miR-3125, and miR-2116. For PDK1 SNPs, the rs1530865 and rs2357637 interaction were assessed with miR-3916, miR-3125 and miR-2116 to determine their risk alleles and their regulation by miRNAs. We found that all the prostate cell lines were only expressing the risk alleles (homozygous GG and CC) for both SNPs. Therefore, we selected some ovarian cancer cell lines for genotyping and found that the ovarian cancer cell lines, CAOV3, OV90 and OVMZ6, were homozygous for the risk allele (GG, CC) and heterozygous (GC, AC) and homozygous for the minor allele (CC, AA), respectively. Interestingly, these SNPs are not previously reported to be associated with the risk of ovarian cancer. We identified that miR-3916 and miR-3125 interacted with the C allele for the rs1530865 SNP and miR-2116 with the A allele of rs2357637 and that overexpression of these miRNAs results in reduced expression of PDK1 protein in OVMZ6 but not in CAOV3 cells. This transition of base pairs in the 3′ UTR of PDK1 disturbed miRNA binding in the PDK1 gene.

Further, overexpression of these predicted miRNAs in LNCaP cells, which expresses the homozygous risk genotype for both SNPs, did not affect PDK1 protein expression. These findings revealed that the 3′-UTR SNPs, rs1530865 and rs2357637, modulate PDK1 gene expression by altering miRNA target binding affinity. We previously discovered the miRSNPs in PDK1 to be associated with the risk of prostate cancer. A crucial next step was to determine the molecular mechanism of this association, which is validated in our current study. Reporter assay to show the allele-specific molecular effects on binding was conducted in the prostate cancer cell line, LNCaP, which highly supported our *in vivo* data from the clinical samples in our previously published study. We used ovarian cancer cell lines as additional models.

PDK1 targeting miRNAs have been reported in other cancers as well. For example, miR-3916 is upregulated in conjunctival melanoma-specific for stage-T1 and associated with an increased risk of local recurrence [40]. miR-3125 is down-regulated in glioblastoma and associated with a poorer prognosis [41]. Further, miR-2116 is significantly down-regulated in hepatocellular carcinoma and the surrounding non-tumour tissues compared with the normal liver samples and identified as a biomarker [42]. However, no study has characterised these three miRNAs in prostate cancer.

Given our earlier observation of higher PDK1 expression in androgen-dependent cell lines, we analysed the effects of androgen and anti-androgen on the predicted miRNA’s expression to evaluate the role of androgen in the regulation of miRNA in PCa. miR-3916 and miR-2116 were upregulated with androgen (DHT) and down-regulated with anti-androgen (enzalutamide) treatment. We did not see any effects on miR-3125 by androgen and anti-androgen. Taken together, the results suggest that miR-3916 and miR-2116 are androgen-upregulated miRNAs in PCa cells. miR-3916 is regulated by androgen while functioning as a tumour suppressor in PCa cells. Most of the androgen-upregulated miRNAs behave as oncogenes [43]. However, few studies have shown that androgen-upregulated miRNAs also function as a tumour suppressor in PCa cells [44].

The expression levels of PDK1 in prostate and ovarian tissues were analysed using the Oncomine databases. PDK1 shows increased expression in the aggressive disease of the prostate and ovaries. Based on the statistically significant level and sample size, we selected Arredounai prostate, Lapointe prostate, Grasso prostate, Lapointe prostate, La Tulippe prostate, Tamura prostate, Yoshihara ovarian, Tothill ovarian, Anglesio ovarian and Lu ovarian to check the expression of PDK1 in prostate and ovarian cancers. The expression analysis of PDK1 indicated the vital role of PDK1 in prostate and ovarian tumourigenesis.

Using PDK1 siRNA, we found that suppression of PDK1 abrogates PDK1-mediated migration and invasion. As higher rates of cancer cell migration and invasion are a hallmark of aggressive cancers, our data suggest that PDK1 is a suitable target for therapeutic intervention in prostate cancer and support the clinical development of PDK1 inhibitors for prostate cancer. Further, developing inhibitors against PDK1 may provide a novel strategy to inhibit the progression of metastatic prostate cancer. Several studies suggested that the synergy of migrastatics with anti-proliferative cancer drugs seems to be a promising way to treat metastasis in various cancers [45]. Therefore, the combination of PDK1 inhibitor with anti-proliferative cancer drugs could be an effective option for treating prostate cancer.

Demonstrating the functional consequence of the allele variants is hindered by the availability of allele-specific models of prostate cancer cell lines. In the future, it will be feasible to undertake experiments with the gene-editing of the single base using CRISPR-based technologies, which can be used for clarifying the allele-specific functional roles of miRNAs in vitro and in vivo. Herein, we reported that PDK1 suppression decreased prostate cancer cell migration, invasion and glycolysis. Emerging evidence indicates that targeting PDK1 may represent an efficient strategy against various cancers [11,46]. Our findings provide evidence that PDK1 may contribute to aggressive disease and prostate cancer progression; therefore, PDK1 could be a promising therapeutic target in prostate cancer. However, this premise will need to be tested in prostate cancer xenograft models as essential experimental platforms for preclinical drug design. 

The present study revealed the role of PDK1 in PCa cells and an important association between miRNA–SNP interaction and PCa progression. This newly identified relationship may have therapeutic potential in PCa and may lead to the development of a personalised medicine approach selectively for patients with the PDK1-SNP-specific genotype.

## 4. Materials and Methods

### 4.1. Analysis of PDK1 in the Publicly Available Databases

mRNA microarray datasets of PCa and normal tissue samples were analysed using the publicly available Oncomine microarray gene expression database (www.oncomine.org, accessed on 25 May 2016).

### 4.2. Cell Culture

RWPE-1, LNCaP, 22RV1, PC3, WPMY-1 and DU145 cell lines were purchased from the American Type Culture Collection (ATCC) (accessed on www.atcc.org) whereas C42B, C42 and LAPC4 were kindly provided by Prof. Pamela Russell (Queensland University of Technology, Brisbane, Australia). Cell lines were maintained at 37 °C with 5% CO_2_. WPMY-1, LNCaP, C4-2B, PC3, LAPC4, C4-2, DU145, and 22RV1 cell lines were cultured in RPMI-1640 containing 5% FBS, DUCaP were cultured in RPMI-1640 supplemented with 10% FBS, and RWPE-1 cells were grown in Keratinocyte-SFM. Ovarian cancer cell lines, CAOV3 and OVMZ6, were cultured in RPMI-1640 containing 10% FBS and DMEM with 10% FBS, respectively. Hypoxic experiments were performed within an incubator at 2% O_2_ at 37 °C in a 5% CO_2_ humidified environment.

### 4.3. siRNA Transfection

siRNA transient transfection was carried out with the Lipofectamine^®^ RNAiMAX (Invitrogen, Catalog number 13778150) or Lipofectamine™ 2000 (Invitrogen, Catalog number 11668019) transfection reagent according to the manufacturer’s instructions. siRNAs used in this study included siPDK1 (s10253 and s10254) (Ambion, Catalog number 4390824) and non-targeting siRNA (Ambion, Silencer select no 1 siRNA, Catalog number 4390843). Briefly, the cells were transfected with 10 nM of siRNA and incubated at 37 °C (5% CO_2_) for 72 h.

### 4.4. miRNA Overexpression

For miRNA overexpression experiments, mirVana^®^ miRNA mimic, Negative Control #1 (Ambion, Catalog number 4464058) and miRVana miRNA Mimic (Ambion, Catalog number 4464066) were used in this study. Cells were transfected with 5–60 nM of miRNA and incubated at 37 °C (5% CO_2_) for 48–72 h. The transfection efficiency was confirmed by qRT-PCR and Western blotting in the transfected prostate and ovarian cell lines.

### 4.5. Proliferation Assays

LNCaP cells were seeded in a 96-well plate with a density of 5000 cells/well, and PC3 cells seeded with 10,000 cells/well and transfected with 10 nM of siRNAs on the following day with Lipofectamine Transfection Reagent (Invitrogen). Proliferation assays were assessed using the IncuCyte Zoom (Essen BioScience). Cell proliferation was measured using the Incucyte Software by analysing the occupied area (% confluence) of cell images over time.

### 4.6. Migration Assays

Briefly, PC3 cells (50,000 cells/well) were plated, and cells were grown to confluence for 24 h and treated with an anti-proliferation agent, mitomycin C (10 μg/mL, Sigma), for 2 h before scratching. Scratches were made with a 96-pin wound maker (EB), and cells were treated with an optimised concentration of siRNAs in 50 μL serum-free media. Cell migration assays were performed using a real-time cell imaging system (IncuCyte, Essen Bioscience (EB)). Then, cells were imaged using the IncuCyte. The wound closure was quantified by an integrated metric: relative wound density.

### 4.7. Invasion Assays

PC3 cells (50,000 cells/well) were grown in the image lock plates, and the cells were grown to confluence for 24 h and treated with an anti-proliferation agent, mitomycin C (10 μg/mL, Sigma), for 2 h before scratching. After scratching, cells were treated with siRNA in 50 µL of media. After 60 min, 50 µL of Matrigel (2 mg/mL) was added on top, and the assay plate was kept in an incubator for one hour. Then, 100 µL of media was added to each well and the plate placed into the IncuCyte and images were taken as described earlier.

### 4.8. Metabolic Assay

Glycolytic and mitochondrial activities were measured using a Seahorse XF96 analyser (Seahorse Bioscience). LNCaP cells (10,000 cells/well) were seeded in 80 µL of media overnight in Seahorse XF96 Cell Culture microplates (Agilent). The next day, cells were treated with siRNAs and controls and placed at 37 °C (5% CO_2_) for 72 h. Cellular metabolism was analysed in the SeahorseXF96 flux analyser using Mitostress and Glycolysis Stress Kits (Seahorse Bioscience). Basal oxygen consumption rates (OCR) and extracellular acidification rates (ECAR) were measured for three cycles followed by successive delivery via port injection of the ATP synthase inhibitor, oligomycin (1.2 μM), the mitochondrial uncoupler FCCP (1 μM), and then a combination of rotenone and antimycin A (1 μM). For glycolysis assays, base media was supplemented with 2 mM L-glutamine, and basal ECAR was measured before injection of 10 mM glucose. After glucose injection, glycolytic capacity and glycolytic reserve were assessed after injection of 1 μM oligomycin, and non-glycolytic acidification was assessed by measuring ECAR in the presence of 100 mM 2-deoxy-d-glucose (2-DG).

### 4.9. miRNA Target Reporter Vector Assay

To validate the in-silico predictions for miRNA–SNP affinity, miRNA target luciferase reporter vector assays were performed. Reporter vectors were constructed for the major and minor SNP allele variants for both SNPs, using the pmirGLO Dual-Luciferase miRNA Target Expression Vector (Promega). A total of 50,000 LNCaP cells/well were seeded in a 24-well plate. After 24 h, cells were transfected with 50 ng vector and 60 nM mirVana miRNA Mimics (Life Technologies) using FuGENE transfection reagent (Promega), then analysed 24 h later using the Dual-Luciferase Reporter Assay System (Promega).

### 4.10. Genotyping of Cell Lines

Genomic DNA was isolated using AllPrep DNA/RNA/Protein Mini Kit (Qiagen) according to the instructions of the manufacturer. The rs1530865 SNP region was PCR-amplified using the forward primer 5′-GTGGAAATCTTCGGGTTTCTATAGG-3′ and the reverse primer 5′-GCCGAAGGGTGGCTGGTTCT-3′ and the region containing rs2357637 SNP was amplified by forward primer 5′ AATATTCTCCTCCCCCAAGAAAATT 3′ and the reverse primer 5′-AAACCAACTAGGAAAAAGCTCTCCC-3′. The PCR products were purified using the Wizard SV Gel and PCR Clean-up System (Promega-cat # A9282). PCR products were sequenced at the Australian Genome Research Facility (AGRF).

### 4.11. RNA Isolation and RT-qPCR

Total RNA was extracted from cells using the ISOLATE II RNA Mini Kit according to the standard protocol. Total RNA, 1000 ng, was taken to synthesise cDNA and qPCR performed using the SYBR Green PCR Master Mix (Life Technologies). Expression was normalised using RPL32 as an endogenous control. Relative expression levels were calculated using the comparative CT (Ct) method. The primer sequences for PDK1 were as follows: forward primer 5′-GAGGTGGCGTTCCTTTGAGG-3′ and reverse primer 5′-AACTGCATCTGTCCCGTAACC-3′. Primer sequences for RPL32 were as follows: forward primer 5′-CCCTTGTGAAGCCCAAGA-3′ and reverse primer 5′- GACTGGTGCCGGATGAACTT-3′.

### 4.12. Western Blotting

Total protein was isolated after 72 h of miRNA mimic transfection using RIPA buffer, and 30 μg of total protein was run using 12% resolving polyacrylamide gel. The levels of PDK1 protein were detected using a rabbit monoclonal anti-PDK1 antibody (Cell Signaling Technology-C47H1 #3820), using standard procedures for Western blotting. Western blots were imaged on an Odyssey Imaging System (LI-COR Biosciences) using fluorescently labelled secondary antibodies (Alexa Fluor 680 & 790—Invitrogen).

### 4.13. Androgen and Anti-Androgen Treatment

LNCaP cells were plated in 5% FBS-containing RPMI-1640 medium. The medium was changed to 5% charcoal-stripped serum (CSS) media for 48 h to deprive the cells of androgens, followed by treatment with an optimised concentration of the androgen, dihydrotestosterone (DHT) (10 nM final) and/or the anti-androgen, enzalutamide (10 μM final), for a further 48 h.

### 4.14. Statistical Analysis

All data were analysed using GraphPad prism software. Results with *p* values of less than 0.05 were considered statistically significant. * *p* < 0.05, ** *p* < 0.01, *** *p* < 0.001, **** *p* < 0.0001. Differences in mean values between two groups were statistically confirmed using the *t*-test, and the Tukey test was used as a post-hoc test to identify group differences in variance analysis.

## 5. Conclusions

Our study analysed the role of the PDK1 gene and functional validation of SNPs and associated miRNAs involved in regulating the PDK1 gene as a mediator of prostate cancer aetiology. The findings from this study suggest that studies of miRNAs and their interactions with SNPs could provide valuable insights into the complicated mechanisms of prostate cancer risk and identify suitable molecule pathways for targeted therapy.

## Data Availability

The data presented in this study are available on request from the corresponding author.

## Supplementary Materials

The following are available online at https://www.mdpi.com/article/10.3390/cancers13143582/s1. The uncropped Western blots and molecular weight markers are shown in the Supplementary Materials. Figure S1: Gene expression of PDK1 in solid tissue normal, primary tumour and metastasis from The Caner Genome Atlas (TCGA) dataset, Figure S2: Efficiency screening of PDK1 knockdown in prostate cancer cells. Figure S3: PDK1-mediated migration and invasion of prostate cancer cells. Figure S4: Schematic diagram showing the miRNA-mRNA nucleotides affinities for PDK1 rs1530865 and rs2357637 SNPs with the SNPs underlined and miRNA seed regions, Figure S5: miR-3916, miR-3125 and miR-2116 did not affect PDK1 mRNA expression and PDK1 protein expression in LNCaP cells, Figure S6: PDK1 expression is up-regulated in ovarian cancer tissues. Table S1: Genotyping for rs1530865 and rs2357637 in prostate and ovarian cell lines.
