# Supplementary material for: Allele-Specific MicroRNA-Mediated Regulation of a Glycolysis Gatekeeper PDK1 in Cancer Metabolism
Source: Cancers (Basel). 2021 Jul 17;13(14):3582. doi: 10.3390/cancers13143582 (PMC8304593; doi:10.3390/cancers13143582)
Supplement: Supplementary file 1 [file cancers-13-03582-s001.zip › cancers-1098337-supplementary.pdf]

Supplementary Materials

Allele Specific MicroRNA Mediated Regulation of a Glycolysis Gatekeeper PDK1 in Cancer Metabolism

Sugarniya Subramaniam, Varinder Jeet, Jennifer H. Gunter, Judith A. Clements and Jyotsna Batra

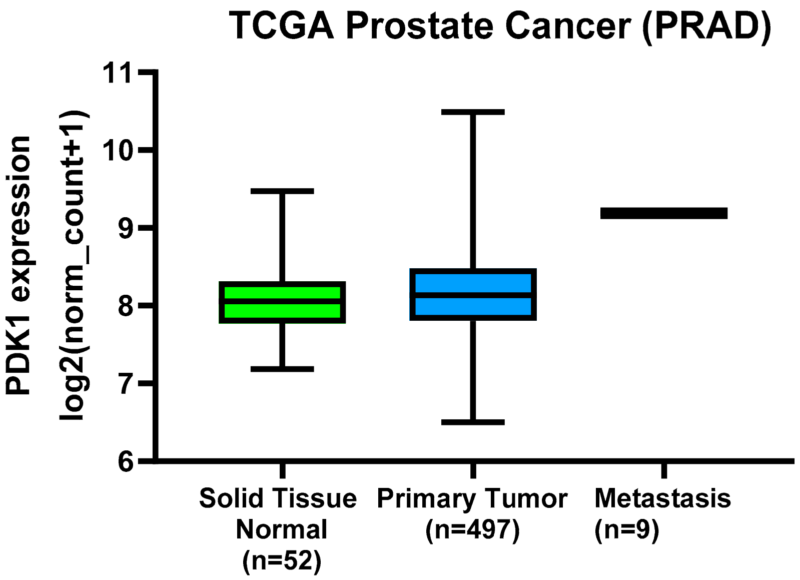

Figure S1. Gene expression of PDK1 in solid tissue normal, primary tumor and metastasis from The Caner Genome Atlas (TCGA) dataset.

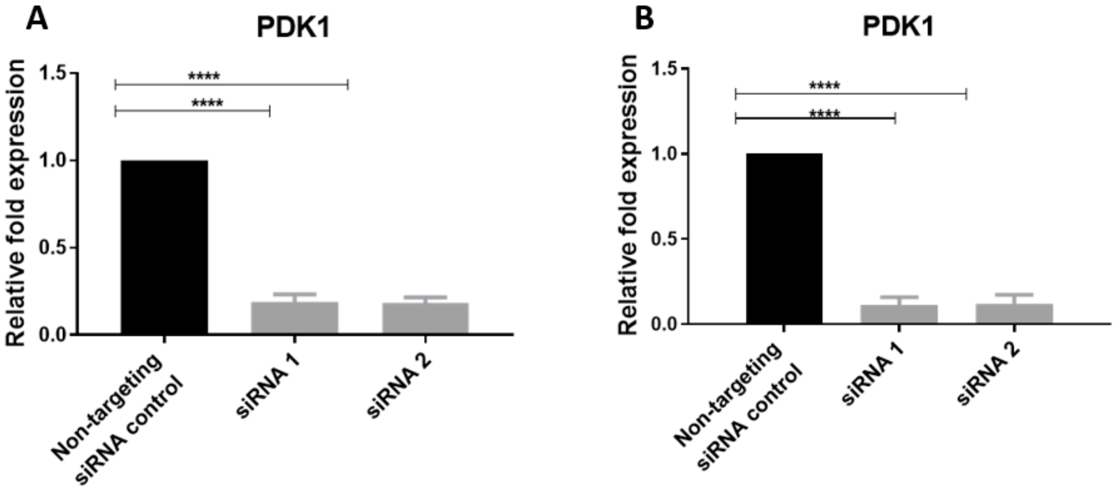

Figure S2. Efficiency screening of PDK1 knockdown in prostate cancer cells. (**A**) LNCaP cells showed 80% knockdown efficiency with siRNAs 1 and 2; (**B**) PC3 cells exhibited 90% knockdown efficiency at the mRNA level. Data presented as Mean ± SEM, $n = 3$ (biological replicates), One-way ANOVA, Tukey's *post-hoc* test **** $p < 0.0001$.

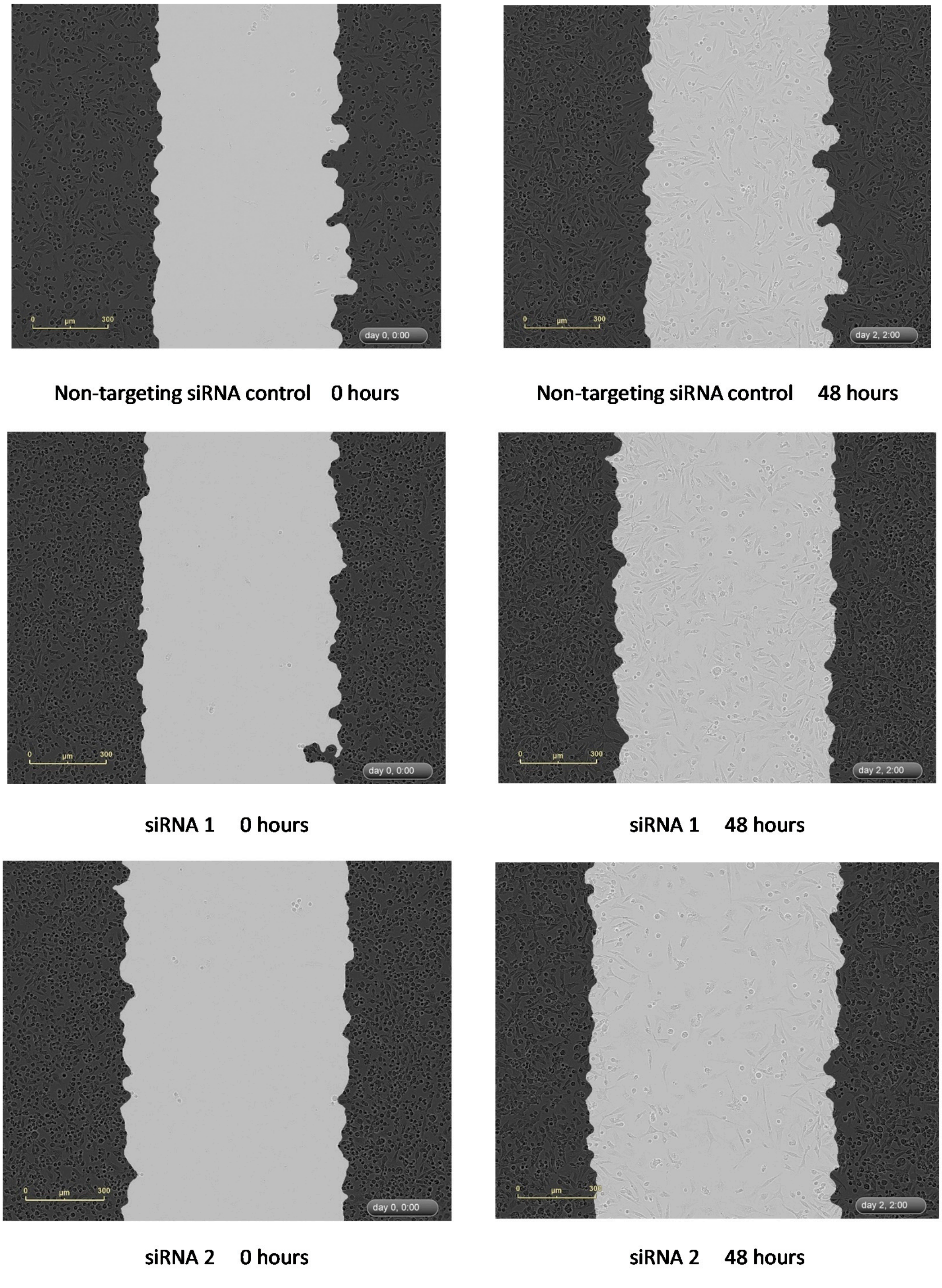
0 μm 300
day 0, 0:00
day 2, 2:00
Non-targeting siRNA control 0 hours
Non-targeting siRNA control 48 hours
siRNA 1 0 hours
siRNA 1 48 hours
siRNA 2 0 hours
siRNA 2 48 hours

(A)

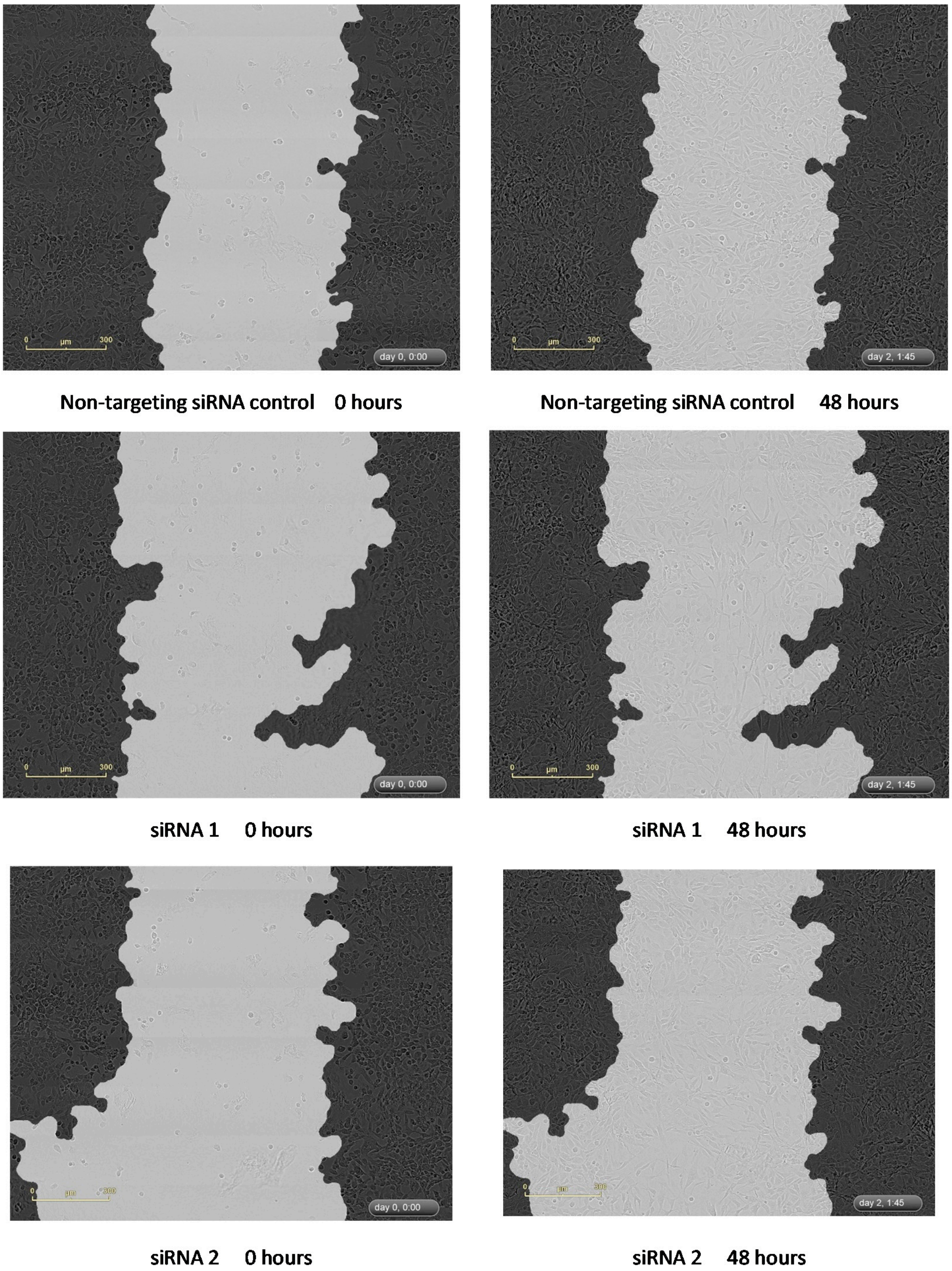

(B)

Figure S3. PDK1-mediated migration and invasion of prostate cancer cells. Representative images are shown for non-targeting siRNA control cells and siRNA treated cells at time points 0 h and 48 h. (**A**) PDK1 suppression reduced the number of percentage of migrating cells in the PC3 wound closure. (**B**) The percentage of cells that invaded through the wounded area reduced with PDK1 suppression.

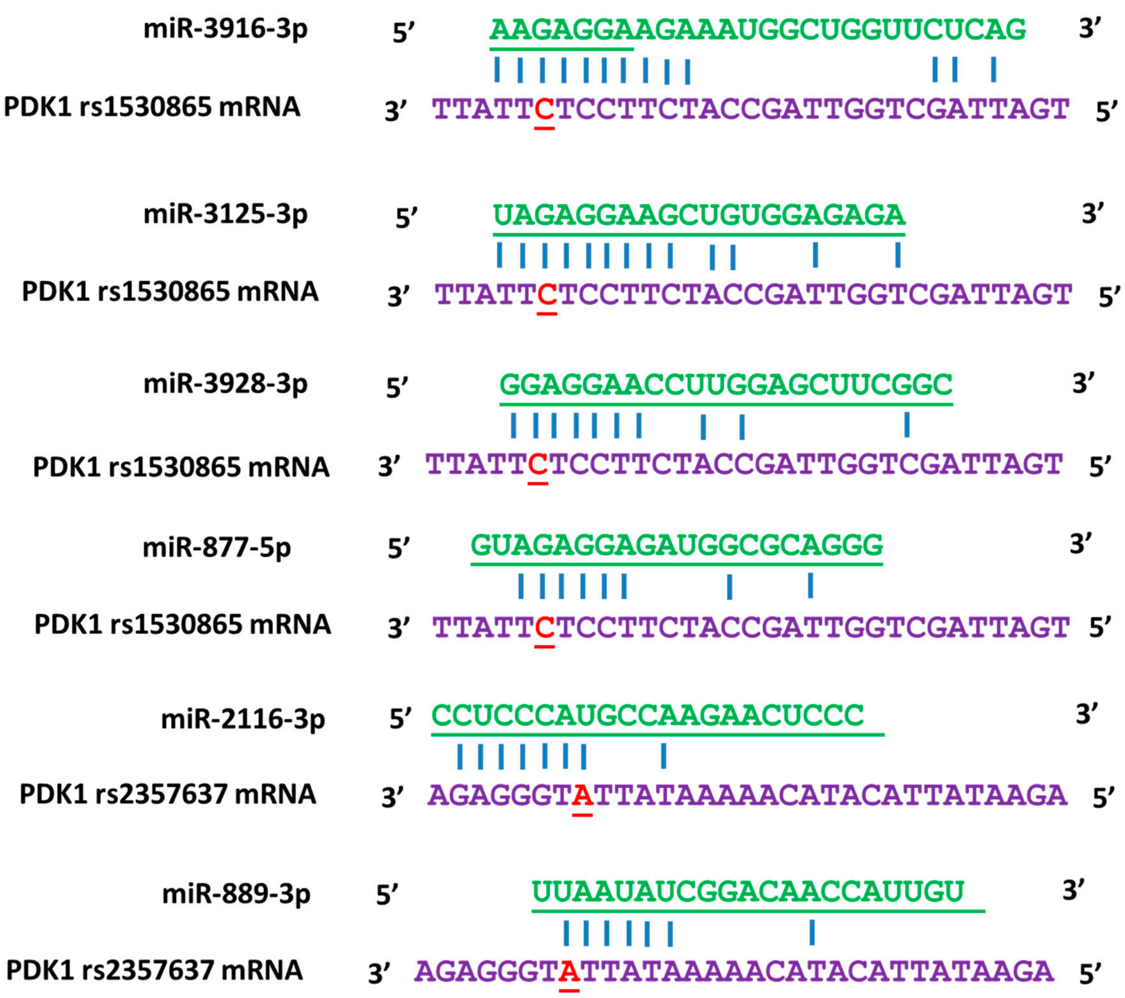

Figure S4. Schematic diagram showing the miRNA-mRNA nucleotides affinities for PDK1 rs1530865 and rs2357637 SNPs with the SNPs underlined and miRNA seed regions.

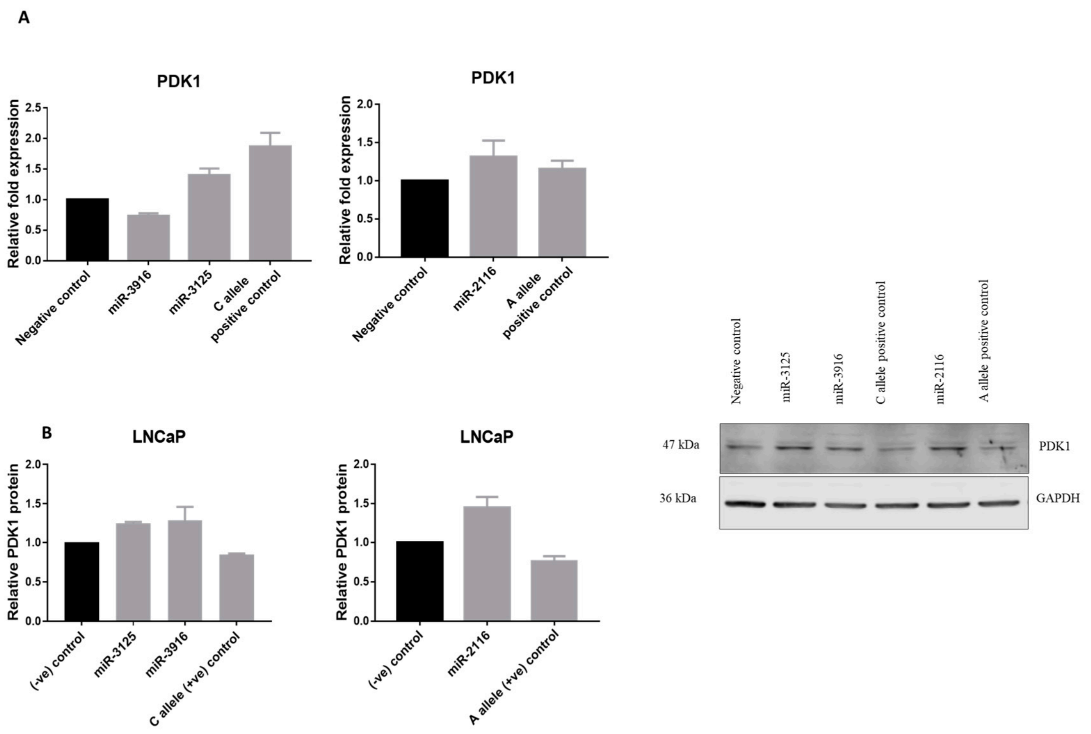

Figure S5. MiR-3916, miR-3125 and miR-2116 did not affect PDK1 mRNA expression and PDK1 protein expression in LNCaP cells. (**A**) Overexpression of miR-3916, miR-3125 and miR-2116 had no effect on PDK1 mRNA level in LNCaP cells. (**B**) miR-3916, 3125 and miR-2116 did not alter PDK1 protein in LNCaP cells. Bar graphs in B indicates densitometry analysis of the western blots. Mean ± SEM, $n = 3$; a representative western blot is shown.

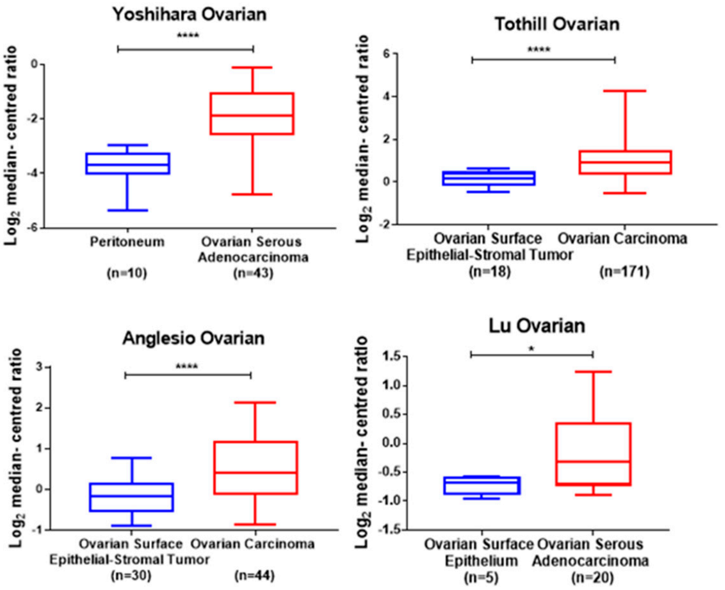

Figure S6. PDK1 expression is up-regulated in ovarian cancer tissues. PDK1 mRNA expression is up-regulated in ovarian carcinoma in four ovarian datasets. (Source-Oncomine, unpaired *t*-test, **** $p < 0.0001$, ** $p < 0.01$, * $p < 0.05$).

Figure 1

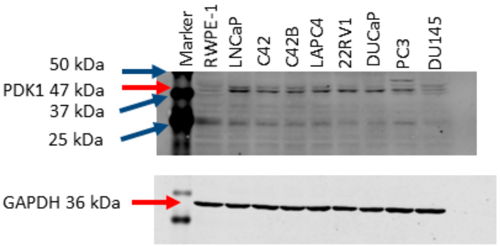

Figure 4

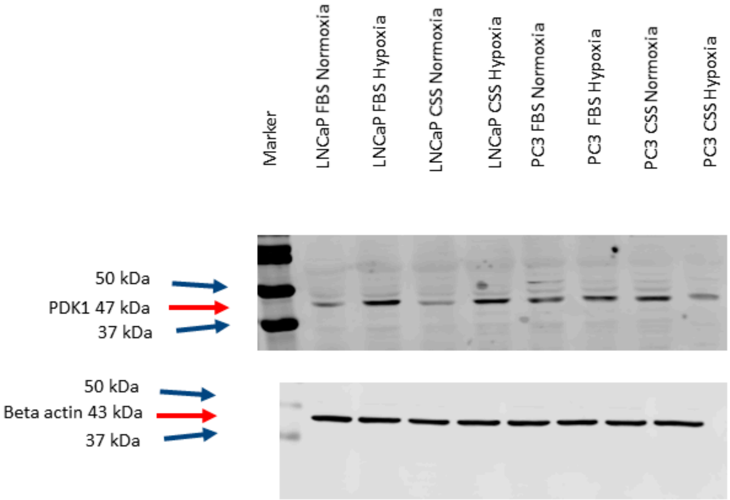

Figure 6B

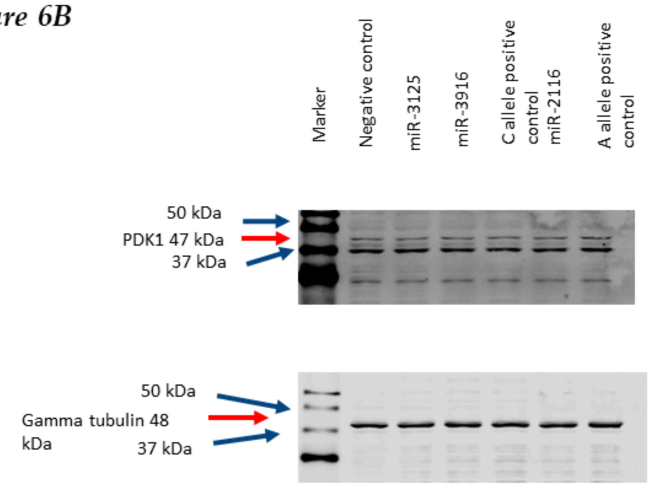

Figure 6D

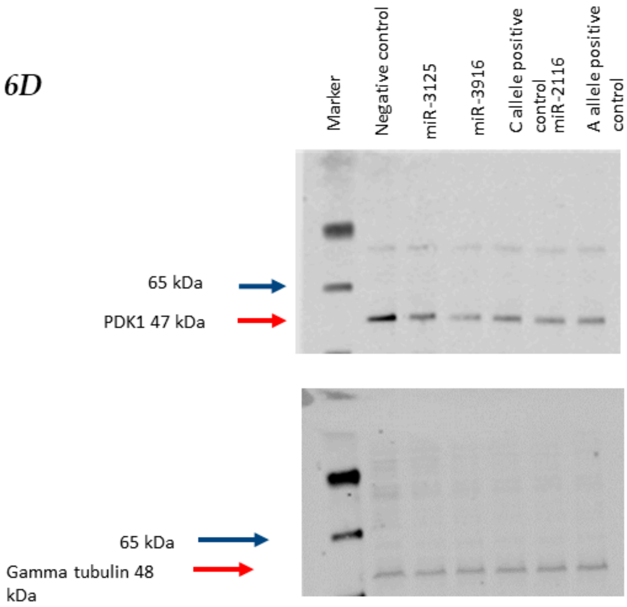

Supplementary Figure 2B

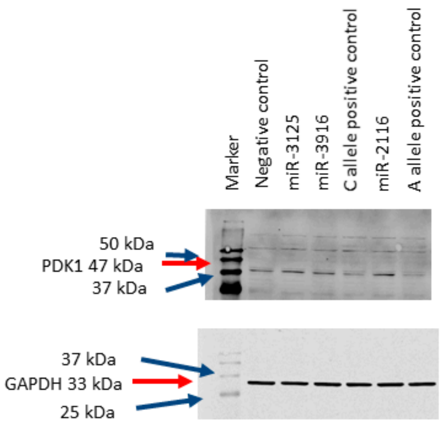

© 2021 by the authors. Licensee MDPI, Basel, Switzerland. This article is an open access article distributed under the terms and conditions of the Creative Commons Attribution (CC BY) license (http://creativecommons.org/licenses/by/4.0/).
